# An analytical comparison of three immunoassay platforms for subpicomolar detection of protein biomarker GAD65

**DOI:** 10.1371/journal.pone.0193670

**Published:** 2018-03-08

**Authors:** Olivier R. Costa, Katrijn Verhaeghen, Sarah Roels, Geert Stangé, Zhidong Ling, Daniel Pipeleers, Frans K. Gorus, Geert A. Martens

**Affiliations:** 1 Department of Clinical Biology, Universitair Ziekenhuis Brussel (UZ Brussel), Brussels, Belgium; 2 Center for Beta Cell Therapy in Diabetes, Brussels Free University (VUB), Brussels, Belgium; 3 Department of Laboratory Medicine, AZ Delta, Roeselare, Belgium; California State University Fresno, UNITED STATES

## Abstract

A disproportional increase of circulating GAD65 within hours from an intraportal islet allotransplantation has been validated as biomarker of beta cell loss and poor functional outcome. More sensitive assays are, however, needed to allow detection of episodes of subtle beta cell loss during late-stage graft rejection or in the peri-onset period of type 1 diabetes. We applied the same sandwich monoclonal antibody couple reactive towards the C- and N-terminus of GAD65 on three advanced immunoassay platforms—the Cytometric Bead Array (CBA, Becton, Dickinson and Company), ElectroChemiLuminescence ImmunoAssay (ECLIA, Meso Scale Discovery) and digital ELISA technology (Single Molecule Array—SIMOA, Quanterix. We then compared analytical performance (linearity, imprecision, limit of detection and functional sensitivity), correlation of results, and practicality. All evaluated techniques showed linearity up to at least 500 ng/dL (76.9 pmol/L). SIMOA achieved the lowest imprecision. The 3 platforms correlate well with each other and could all detect subpicomolar concentrations of GAD65 in plasma, but only SIMOA and CBA could quantify down to that range. SIMOA can achieve the highest sample throughput. The three methods tested allow sensitive detection of GAD65, but SIMOA appears best suited for automated quantification of subpicomolar concentrations.

## Introduction

Several biomarkers have been proposed for ultrasensitive real-time detection of pancreatic beta cell injury *in vivo* [1–3] The biomarker with the longest track record thus far is the type 1 diabetes autoantigen glutamic acid decarboxylase 65kD (GAD65): in rodent models, GAD65 is discharged proportionately to the experimentally-induced degree of beta cell destruction [3]. We demonstrated that, in diabetic patients receiving an intraportal islet allotransplantation, the acute post-transplant plasma GAD65 surge can be used as companion test to monitor massive early graft destruction, impacting long-term outcome [4]. The latter study used an in-house developed sandwich immunoassay for GAD65 using monoclonal antibodies raised towards the C- and T-terminal domains of human GAD65 [5, 6] and time-resolved fluorescence as detection method (TRFIA). The functional sensitivity of this TRFIA, 17.4 ng/dL (2.68 pmol/L) was insufficient to consistently detect GAD65 levels in all recipients of an islet allograft within the hours after implantation, indicating the need for a more sensitive assay [4]. Such assay would also be required to detect more discrete episodes of beta cell loss at later time points. With the use of the same sandwich antibody couple as in our GAD65-TRFIA, we recently developed an enhanced sensitivity Cytometric Bead Array (CBA) assay (Becton, Dickinson and Company), resulting in a 35-fold gain in functional sensitivity, and the detection of beta cell injury up to 24h post-islet implantation [7].

In the present study, we investigated whether the analytical sensitivity of the GAD65 assay could further be improved by implementing the same sandwich antibody couple on two other advanced immunoassay platforms with fluidics and/or detection chemistry designed for high sensitivity: the electrochemiluminescence-based Meso Scale Discovery platform (MSD ECLIA) [8] and digital ELISA technology (Single Molecule Array—SIMOA, Quanterix) [9]. In addition to a CLSI guideline-based analytical evaluation focusing on test sensitivity [10], we also evaluated their practicality and sample throughput.

## Materials and methods

### Monoclonal antibody couple for GAD65 sandwich immunoassays

The GAD65 antibody couple used throughout this study consisted of a capture antibody (clone GAD6, kindly provided by Dr. D. Gottlieb, St. Louis, MO, USA) raised in mouse and reactive to the C-terminal region of human and rat GAD65 [5], and a detection antibody (clone mAb144, kindly provided by Dr. C. Hampe, Seattle, WA, USA) raised in mouse and reactive to the N-terminal region of human and rodent GAD65 [6]. Biotinylation of antibodies was performed with the use of EZ Link^™^ NHS-Biotin (Thermo Fisher Scientific Inc.) according to the manufacturer’s instructions (https://tools.thermofisher.com/content/sfs/manuals/MAN0011206_EZ_NHS_Biotin_Reag_UG.pdf).

### CBA assay

The GAD6 capture antibody was covalently attached to CBA functional beads (E7, Becton Dickinson) with sulfosuccinimidyl 4-(N-maleimidomethyl) cyclohexane-1-carboxylate (Sulfo-SMCC) chemistry according to the manufacturer’s protocol (https://www.bdbiosciences.com/documents/CBA_FunctionalBeadConjugation_Set_Manual.pdf). The assay was performed using a high-sensitivity buffer kit (Becton, Dickinson and Company) and signal was read on a FacsAria^™^ (Becton, Dickinson and Company) device according to the procedure previously described [7].

### MSD ECLIA assay

Ruthenium was covalently attached to the mAb144 detection antibody using an MSD-GOLD^™^ Sulfo Tag NHS-Ester conjugation kit (Meso Scale Discovery, MSD) through N-(3-dimethylaminopropyl)-N’-ethylcarbodiimide hydrochloride (NHS) chemistry according to the manufacturer’s protocol (https://www.mesoscale.com/~/media/files/handouts/msd%20gold%20sulfotag%20conjugation%20quick%20guide.pdf) in a 1:12 proportion to make SulfoTag mAb144.

Blocking solution containing 5 g/dL Bovine Serum Albumin (MSD) was added (150 μL) to each well of an MSD GOLD^™^ 96 Small Spot Streptavidin-coated plate (MSD) and was incubated for 1 hour at room temperature, on a platform shaker (100 strokes/minute). All subsequent incubation steps were performed under this type of constant shaking. After 3 wash cycles with PBS (Sigma-Aldrich Co) supplemented with Tween-20 (Merck) 0.05% (v/v) (PBS-T), 30μL of 0.72 μg/mL biotinylated mAbGAD6 in PBS was added to the wells and incubated for 1 hour at room temperature. After 3 wash cycles, 25μL of sample was added to each well and plates were incubated overnight at 4°C. Wells were washed 3 times, and 25μL of 0.32 μg/mL of SulfoTag mAb144 in PBS-T supplemented with 1g/dL BSA was incubated for 2 hours at room temperature. After another 3 wash cycles, 150 μL of “2x”-concentrated Read Buffer (MSD) was added to each well. The plates were read with a MESO QuickPlex SQ 120 imager that uses electrochemiluminescent SulfoTag labels which generate light when stimulated by electricity.

### SIMOA assay

The capture antibody was covalently attached to Homebrew Carboxylated Paramagnetic Beads (Quanterix) with 1-ethyl-3-(3-dimethylaminopropyl) carbodiimide (EDAC) chemistry according to the manufacturer’s protocol.

Samples were prediluted 25:90 in Homebrew Sample/Detector Reagent (Quanterix) in Microwell^™^ V96 polypropylene plates (Nunc). Homebrew Bead Reagent was prepared by diluting stock bead solution 16:1000 in Sample/Detector Reagent. Homebrew Detection Reagent was a 0.30 μg/mL solution of biotinylated mAb144 in Sample/Detector Reagent. Homebrew streptavidin-β-galactosidase (SBG) reagent was prepared by diluting SBG Concentrate (Quanterix) to a final concentration of 11.6 g/dL in SBG Diluent (Quanterix). Undiluted resorufin-D-galactopyranoside (RBG) (Quanterix) was used as dye. All incubations and washing steps were performed on the fully automated SIMOA HD-1 (Quanterix) analyzer according to the SIMOA 2.0 protocol (2-step fashion) in single-analysis cuvettes. The first incubation consisted of 90 μL of prediluted sample, 35 μL of Homebrew Bead Reagent and 30 μL of Homebrew Detection Reagent during 35 minutes and 15 seconds. This was followed by an automatic washing step and a second incubation of 5 minutes and 15 seconds with Homebrew SBG reagent and RBG. After a final washing step, the beads were transferred to the SIMOA Discs, followed by signal detection on board of the analyzer.

### Samples

All materials were prepared in human plasma (2.6 mL K_3_-EDTA monovette tubes [Sarstedt], supplemented with 1.2 μg/mL Aprotinin [Stago]), obtained from a healthy donor after informed consent. Standard curves were obtained by spiking human recombinant GAD65 (Diamyd Medical) to plasma devoid of GAD65. Lysate samples were prepared by spiking human plasma with a lysate of human pancreatic islet cells. Human pancreatic islet cells were provided by the Beta Cell Bank at Universitair Ziekenhuis Brussel (UZB) with approval of the local Institutional Review Board (UZB Ethical Committee; BUN 143201213515 and BUN14320109289). Aliquots were prepared for single use and stored at -80°C for subsequent evaluation of the three techniques.

### Analytical validation

For each assay, a standard curve was analyzed in triplicate. Lysate samples were measured in duplicate and results were obtained by linear interpolation to this standard curve. For the determination of Limit of Blanks (LoB) and Limit of Detection (LoD), the assay was calibrated in the 0–10 ng/dL (0–1.54 pmol/L) range. For the analysis of lysate samples and for determining the Limit of Quantification (LoQ), it was calibrated in the 0–100 ng/dL (0–15.4 pmol/L) range.

Linearity was evaluated according to the CLSI guideline EP06-A [11] by triplicate measurement in human plasma, spiked with various amounts of human recombinant GAD65 (n = 10) to cover a concentration range between 5 and 500 ng/dL (0.77–76.9 pmol/L). Linearity was assessed by linear regression analysis. Within-run, between-day and total variation were estimated according to the CLSI guideline EP5-A [12]: a low and a high concentration of GAD65 were prepared for each evaluation by spiking human plasma with a lysate of human pancreatic islet cells and determined on 10 different days. As the instruments were not present at the same time in the lab, different preparations were used.

Analytical sensitivity was determined according to the CLSI guideline EP17-2A [10, 13]: to determine functional sensitivity, plasma samples spiked with increments of a human islet cell lysate were measured in duplicate on 10 different days and the %CV plotted as function of GAD65 concentration to graphically determine the analyte concentration at which 20% CV is reached. LoB (mean of blank + 1.645SD) and LoD (mean of blank + 3SD) were determined by repeated (n = 20) measurement of a blank plasma sample from a healthy donor on 3 different days. Method comparison between different platforms and CBA was performed by Deming regression analysis on samples of human islet cell lysate spiked in plasma. Split sample analysis was used to compare GAD65 concentrations obtained with CBA and the two other techniques, only taking into account samples with GAD65 values above the functional sensitivity of the respective assays. The degree of correlation was assessed with 2-tailed Spearman correlation test and by determining R^2^ and the standard deviation of the residuals (Sy.x).

Statistical analysis was performed with Prism 5 (GraphPad) software using the statistical tests specified above.

## Results

### Linearity

**Fig 1:**
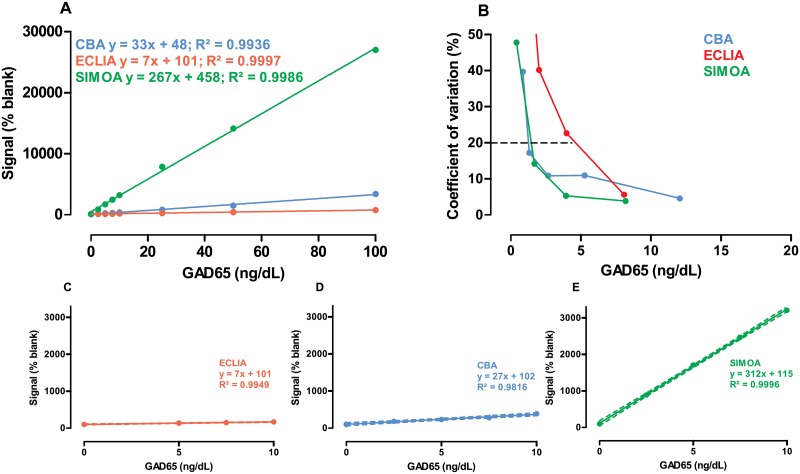
Linearity, slope and Limit of Quantification. (A) Linearity of the raw data signal expressed as percent of blank for CBA (blue), SIMOA (green) and ECLIA (red) in the 5–100 ng/dL (0.77–15.4 pmol/L) GAD65 range. Slope, intercept and R^2^ are shown in corresponding colors. (B) Determination of LoQ as the GAD65 concentration at which the between-day coefficient of variation reached 20% (indicated by dashed line) as assessed from n = 10 independent runs; the interpolated LoQ is shown in Table 1. (C) Linearity in the 5–10 ng/dL (0.77–1.54 pmol/L) range for ECLIA (C). (D-E) linearity in the 2.5–10 ng/dL (0.38–1.54 pmol/L) range for CBA (D) and SIMOA (E). Dashed lines: 95% confidence interval.

The linearity of the GAD65 CBA, MSD ECLIA and SIMOA assays was excellent (R^2^ >0.99) in the 5–100 ng/dL (0.77–15.4 pmol/L) GAD65 range (Fig 1A), with a dynamic range from 5 ng/dL (0.77 pmol/L) up to 500 ng/dL (76.9 pmol/L) (DOI 10.17605/OSF.IO/XWYB2). Also in the lower GAD65 concentration range, linearity was excellent for all 3 assays (Fig 1C–1E).

**Table 1:** Analytical sensitivity of GAD65 immunoassay on three different analytical platforms.

		CBA	MSD ECLIA	SIMOA
Analytical sensitivity		ng/dL (pmol/L)	ng/dL (pmol/L)	ng/dL (pmol/L)
Limit of Blank	mean_blank_ + 1.645(SD^a^_blank_)	0.49 (0.075)	1.68 (0.258)	0.25 (0.038)
Limit of Detection	mean_blank_ + 3(SD^a^_blank_)	0.79 (0.122)	3.51 (0.540)	0.49 (0.075)
Limit of Quantification	GAD65 at CV^b^ = 20%	1.27 (0.195)	4.76 (0.732)	1.46 (0.225)

^a^ standard deviation;

^b^ coefficient of variation

### Imprecision

SIMOA achieved the lowest (total) imprecision both at low and high GAD65 levels (Table 2). MSD ECLIA performed better than CBA at the high GAD65 level, but at the lower GAD65 level CBA was slightly more precise than MSD ECLIA.

**Table 2:** Imprecision of GAD65 immunoassay on three different analytical platforms.

Imprecision	CBA		MSD ECLIA		SIMOA	
	SD^a^	CV^b^	SD^a^	CV^b^	SD^a^	CV^b^
	ng/dL (pmol/L)	%	ng/dL (pmol/L)	%	ng/dL (pmol/L)	%
**High GAD65**						
Overall mean	69.2 (10.6)^c^		63.6 (9.78)^c^		44.1 (6.78)^c^	
Within-run	6.8 (1.05)	10.5	3.8 (0.58)	6.0	2.1 (0.32)	4.8
Between day	2.7 (0.41)	3.9	2.3 (0.35)	3.6	2.2 (0.34)	4.9
Total	6.5 (1.00)	9.4	4.0 (0.62)	6.3	2.8 (0.43)	6.4
**Low GAD65**						
Overall mean	11.5 (1.77)^c^		8.0 (1.23)^c^		8.0 (1.23)^c^	
Within-run	1.3 (0.20)	11.5	0.1 (0.02)	1.4	0.1 (0.02)	0.8
Between day	0.6 (0.10)	5.6	1.0 (0.15)	12.9	0.3 (0.05)	3.5
Total	1.3 (0.20)	11.2	1.0 (0.15)	13.0	0.3 (0.05)	3.6

^a^ standard deviation;

^b^ coefficient of variation;

^c^ overall mean of sequentially prepared and determined samples; calculations according to CLSI protocol EP17-A2 [10].

### Method comparison

**Fig 2:**
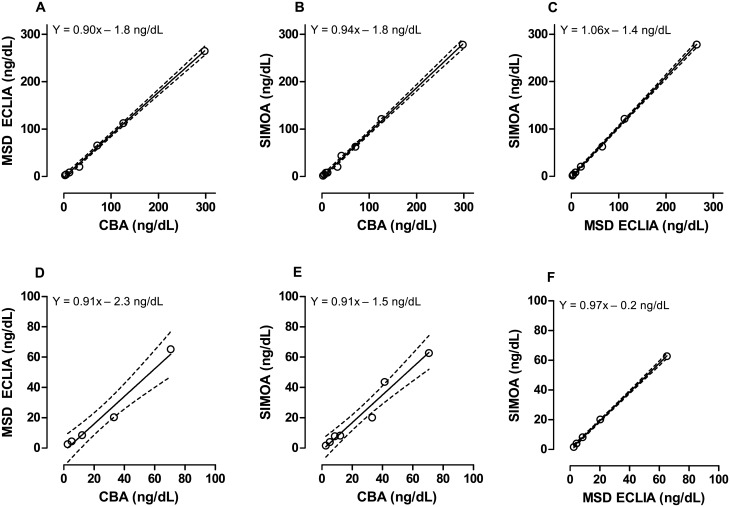
Comparison of CBA, MSD ECLIA and SIMOA methods using lysate samples. The techniques were compared two by two, and slopes and y-intercepts (including 95% CI) were computed for GAD65 values < 298 ng/dL (45.8 pmol/L) (panels A-C) and < 70 ng/dL (10.8 pmol/L) (panels D-F) level. (A) CBA/MSD ECLIA: slope (95%CI) = 0.90 (0.86–0.94); y-intercept (95%CI) = -1.8 (-6.8–3.1) ng/dL; R^2^ = 0.998. (B) CBA/SIMOA: slope (95%CI) = 0.94 (0.90–0.99); y-intercept (95%CI) = -1.81 (-6.51–2.88) ng/dL; R^2^ = 0.997. (C) MSD ECLIA/SIMOA: slope (95%CI) = 1.06 (1.03–1.09); y-intercept (95%CI) = -1.36 (-4.79–2.06) ng/dL; R^2^ = 0.999. (D) CBA/MSD ECLIA: slope (95%CI) = 0.91 (0.63–1.19); y-intercept (95%CI) = -2.3 (-12.3–7.7) ng/dL; R^2^ = 0.972. (E) CBA/SIMOA: slope (95%CI) = 0.91 (0.70–1.13); y-intercept (95%CI) = -1.5 (-8.9–5.8) ng/dL; R^2^ = 0.959. (F) MSD ECLIA/SIMOA: slope (95%CI) = 0.97 (0.94–1.00); y-intercept (95%CI) = -0.23 (-1.28–0.81) ng/dL R^2^ = 0.999. Dashed lines: 95% confidence interval.

GAD65 levels measured in lysate samples by MSD ECLIA and SIMOA, correlated well with those obtained by CBA as indicated by p < 0.001 for Spearman test for both platforms, high R^2^ values (0.998 for both) and low Sy.x values (4.79 ng/dL [0.74 pmol/L] and 4.80 ng/dL [0.74 pmol/L], for MSD ECLIA and SIMOA, respectively). Results obtained with MSD ECLIA and SIMOA were even better correlated, as indicated by p < 0.0001 (Spearman test), R^2^ > 0.999 and a low Sy.x value of 2.79 ng/dL (0.43 pmol/L) (Fig 2).

### Sensitivity

The ability of an assay to qualitatively discern analyte-devoid blank samples from samples with low analyte levels, depends on the noise detected in the blank samples (LoB), the gain of raw signal by low analyte levels and the imprecision by which low analyte levels are measured (LoD). SIMOA consistently achieved the highest gain in raw signal, 4-times higher than CBA and 18-times higher than ECLIA (Fig 1C–1E). SIMOA and CBA both showed very limited noise in analyte-devoid samples, resulting in Limits of Blank in the femtomolar range (Table 1). While showing slightly less noise in analyte-devoid samples, SIMOA showed a slightly higher inter-assay variation on its slope in this low range (Fig 1C–1E). CBA and SIMOA outperformed ECLIA: although the latter method achieved a good overall sensitivity with LoD in the subpicomolar range, it produced a 10-times higher noise level in blank plasma and a 5-times lower precision at low GAD65 levels. Finally, we determined the assays’ LoQ, defined here as the GAD65 concentration at which a coefficient of variation of 20% is observed (Fig 1B, Table 1): again, CBA and SIMOA were comparable with a LoQ close to 1ng/dL (0.15 pmol/L).

### Comparison of practicality

Finally, we compared cost and throughput of the different immunoassay platforms (Table 3). CBA and SIMOA have a similar reagent cost, which is higher than for MSD ECLIA. On the other hand, SIMOA requires less hands-on time (and thus labor cost) compared to the other two platforms, which makes it more suitable for the analysis of larger sample batches. All platforms have multiplexing capability, which means other proteins of interest can be assayed in parallel as a biomarker panel for the evaluation of beta cell damage [14] or function (e.g. C-peptide), without the need of larger sample volumes.

**Table 3:** Comparison of the practical aspects of the immunoassay platforms tested for the analysis of a 96-well plate.

Method characteristic	CBA	MSD ECLIA	SIMOA
Approximate assay run time	8–10 h	12–20h (ON)	2–3 h
Approximate hands-on time	4.0–5.0 h	2.0–3.0 h	1.0–1.5 h
Automation	No	Optional	Default
Approximate cost of reagent per well	++	+	++
Multiplexing capacity	Yes	Yes	Yes

ON = Overnight incubation; Cost per well: +: 2$—3$; ++: 3$—5$

## Discussion

The strength of this method comparison study is that we implemented the same antibody couple on three advanced immunoassay platforms and tested it on a common sample set, allowing an objective analysis of the relative sensitivities of the underlying technologies. All three platforms were designed for high sensitivity due to specific signal amplification chemistries (electrochemiluminescence for ECLIA, enzymatic for SIMOA and fluorescent enhancement for CBA), solid phase (beads in CBA and SIMOA) or microfluidics (digital assay format unique to SIMOA). In our hands, all three platforms achieved excellent sensitivity, confidently detecting GAD65 concentrations in the femtomolar range in plasma matrix. Intrinsically, the digital SIMOA technology appears the most sensitive though followed closely by CBA: SIMOA showed the highest gain in raw signal, and the lowest noise in blank samples. This results in a remarkably steep slope, compared to the other two platforms. Nevertheless, the excellent robustness of the CBA, with lower inter-assay imprecision at low analyte levels, resulted in comparable functional sensitivities for both platforms. This finding illustrates the importance of taking both steepness of curve and imprecision into account when evaluating sensitivity of an analytical platform. SIMOA and CBA clearly outperformed ECLIA, suggesting that the plate-based solid phase setup of the latter has limitations. To circumvent these, a modification of the procedure could be envisaged whereby capture antibody and sample are incubated in the liquid phase before being added to the solid phase for measurement [15]. For studies on the immunogenicity of monoclonal antibody therapeutics, such a procedure limits the number of washing steps applied to the assay, and thus allows to detect low affinity antibodies, that would otherwise be washed away [15]. Although not tested here, we believe such an adaptation would not be beneficial to the MSD ECLIA platform, as the capture antibody used displays a high affinity [16] and a reduction of the number of washing steps would not be beneficial for robustness. The GAD65 assay implemented on CBA and SIMOA can be considered extremely sensitive: not only are both assays 5 to 8 times more sensitive than previously described GAD65 immunoassays [17, 18], but with their LoD around 0.7 ng/dL (0.11 pmol/L), they are only 3 times less sensitive than the Singulex Erenna^®^ cTnI assay, which is considered one of the most sensitive immunoassays ever developed [19].

Our study also showed limitations. It did not include some other technologies with acclaimed high sensitivity: besides the aforementioned Single Molecule Counting Erenna^®^ technology, also proximity ligation assays [20] can achieve extreme sensitivity due to PCR-based signal amplification: recently a proximity ligation assay was reported [21] to detect GAD65 concentrations as low as 0.065 ng/dL (0.01 pmol/L). Though theoretically feasible, this reported detection limit was not based on a thorough method evaluation using internationally accepted guidelines, and thus did not take into account inter-assay variability and lack of robustness as important source of variation, in particular for DNA amplification-based chemistries. Other limitations were the absence of certified plasma-based control material for GAD65, impeding verification of trueness of this biomarker, and the lack of clinical verification of respective platforms on patient samples, e.g. the long-term follow up after islet transplantation, due to scarcity of these samples.

We conclude that the three analytical platforms tested allow detection of GAD65 in the subpicomolar range. The SIMOA platform seems most appropriate to further investigate low-grade beta cell loss because its functional sensitivity equals that of CBA while being best suited for automated analysis of large sample batches. Further studies should be initiated to collect these samples to further elucidate the still largely enigmatic timing and extent of beta cell destruction during prolonged follow-up post-islet transplantation, and ultimately during earlier (pre)clinical stages of type 1 diabetes.
